# Universal tumor screening for Lynch syndrome: Perceptions of Canadian pathologists and genetic counselors of barriers and facilitators

**DOI:** 10.1002/cam4.2182

**Published:** 2019-05-17

**Authors:** Elizabeth Dicks, Daryl Pullman, Ken Kao, Andrée MacMillan, Gabrielle S. Logan, Charlene Simmonds, Holly Etchegary

**Affiliations:** ^1^ Faculty of Medicine Memorial University St. John’s NL Canada; ^2^ Immunohistochemistry Laboratory Eastern Regional Health Authority St. John’s NL Canada; ^3^ Provincial Medical Genetics Program Eastern Regional Health Authority St. John’s NL Canada

**Keywords:** Colorectal Neoplasms, tumor screening, genetic counseling, Lynch syndrome, pathologists

## Abstract

**Background:**

People at risk of developing hereditary cancers associated with Lynch Syndrome (LS) can be identified through universal screening of colorectal tumors. However, tumor screening practices are variable across Canada and few studies explore the perspectives of genetic counselors and pathologists about tumor screening. This study was conducted to better understand the barriers and facilitators of implementing universal tumor screening in health centers across Canada.

**Methods:**

An online survey about tumor screening programs was administered to genetic counselors and pathologists across Canada through communication channels of professional organizations. It was hosted on SurveyMonkey and accessible from October 2016 to March 2017.

**Results:**

Barriers to tumor screening included a lack of sustainable resources, including funding and genetic counselors. Respondents strongly identified the need for a coordinated, interdisciplinary approach to program planning with the “right people at the table.” Respondents currently with a screening program provided advice such as carefully designing the program structure, developing patient and family follow‐up protocols, and ensuring adequate resources (funding, staff, training for providers) were available prior to program initiation.

**Conclusion:**

There is no national approach to universal tumor screening in Canada. However, future efforts can be informed by the experiences of those centers that have already created a universal tumor screening program for LS. These data suggest the need for an interdisciplinary approach, initial and sustained funding, and careful advanced planning of program structures and policies.

## 1 INTRODUCTION

As the second most common cancer in Canada, colorectal cancer (CRC) accounted for 13% of all cancers in 2017.^1^ Lynch Syndrome (LS), an autosomal dominant genetic condition, is the most common heritable cause, accounting for 3%‐5% of all CRCs.^2^, ^3^

Lynch Syndrome not only significantly increases the lifetime risk of CRC, but also pancreatic, stomach, and other malignancies, with an appreciably higher lifetime risk of endometrial cancer for female carriers.^4^, ^5^, ^6^ Given these substantial lifetime cancer risks, early identification of high‐risk individuals is critically important for cancer prevention.

Traditionally, patients with CRC evaluated for LS were identified using family history information and age of cancer onset as outlined in the Amsterdam criteria and Bethesda guidelines.^7^, ^8^, ^9^, ^10^ However, because of their limited sensitivity and specificity, they have significant drawbacks.^11^ Many mutation carriers are not individuals who would have been tested for LS according to these criteria, and some who do meet the criteria are improperly diagnosed.^12^, ^13^, ^14^ As a result, some high‐risk families remain unidentified and are unable to avail early prevention strategies.^11^, ^15^, ^16^

The underpinning cause of LS is loss‐of‐function mutations in any of the genes that encode the DNA mismatch repair (MMR) proteins MLH1, MSH2, MSH6, or PMS2.^17^ As such, recent strategies to better identify high‐risk individuals include “reflex testing” the tumors of patients with CRC for MMR deficiency using either immunohistochemistry (IHC) or microsatellite instability (MSI).^15^, ^16^, ^18^

The better sensitivity of reflex tumor testing to identify possible cases of LS led to recommendations of universal tumor screening for all individuals with CRC, regardless of age or family history.^15^ This initial call was followed by further support from the US Multi‐Society Task Force on CRC and other national organizations and initiatives.^11^, ^19^

Despite the calls for universal screening programs, however, there is variable uptake of reflex testing and heterogeneity in practice.^20^, ^21^, ^22^, ^23^, ^24^ In the UK, for example, screening for LS in the National Health Service is “patchy and inconsistent” with a variety of screening protocols in use across institutions.^25^ In the US, driven in part by the Healthy People 2020 policy objective “to increase the proportion of persons with newly diagnosed CRC who receive genetic testing to identify Lynch syndrome,” universal screening has been implemented regionally by over 30 health systems and on a statewide basis in Ohio.^2^, ^26^ Nonetheless, LS screening protocols are more likely in research and academic centers, and less likely in community hospitals.^20^, ^27^ A recent systematic review of existing screening pathways for LS^28^ revealed only five sites with structured and permanent screening protocols: three in the US, one in Australia, and one in Europe. Of these, three followed a universal screening approach including all patients with CRC. The Australian pathway included CRC patients <60 years, while the Californian site included CRC patients <50 years. In Canada, there is no standardized and integrated approach to the identification of patients with LS.^22^, ^23^, ^29^, ^30^ Rather, identifying potential cases of LS depends largely on individual clinicians, leaving some families with LS unidentified.^23^

Barriers to implementing tumor screening programs for LS include a lack of financial and human resources, low awareness of LS and tumor screening, a lack of education for healthcare providers, and a general lack of information on exactly how to implement the program, complicated by variable program structures and protocols.^16^, ^19^, ^23^, ^31^, ^32^

To inform discussions about universal tumor screening programs for LS, this study presents the results of an online survey of Canadian pathologists and genetic counselors. Barriers and facilitators to implementing a tumor screening program for LS from the viewpoint of those currently with, and without, a screening program at their centers are described.

In this province, universal tumor screening could positively impact health outcomes. Newfoundland and Labrador (NL) has the highest incidence of CRC in Canada, and the highest CRC mortality rate.^33^ Statistics from the Canadian Cancer Society show NL with the highest stage‐specific, age‐standardized incidence rate of CRC of around 20 per 100 000 for Stage II and III colon cancer.^1^ With its small population (~528 000), CRC represents a significant public health burden for the province.

The purpose of this study was to explore the barriers and facilitators related to implementing a tumor screening program for LS. There is little practical information in the literature regarding how centers have proceeded with implementing a tumor screening program. The objective of this paper is to reveal identified barriers and advice about implementing tumor screening programs for LS in the hope that other centers may learn from those with experience.

## 2 METHODS

Ethical approval was obtained from the Health Research Ethics Board in St. John's, Newfoundland and Labrador, Canada (Ref # 16.062).

### 2.1 Survey development and content

The full description of survey content and iterative development is described elsewhere.^30^ Briefly, two descriptive, online surveys were developed for genetic counselors (38 items) and pathologists (36 items). Additional items for genetic counselors probed follow‐up protocols in the event of an abnormal tumor screen. Items from prior published surveys^21^, ^22^ were used or slightly modified for current surveys (eg, which tumors were routinely screened, criteria for screening, type of test used, ordering healthcare professional), with items measuring attitude toward tumor screening and consent practices crafted by the research team.

In this paper, items measuring barriers to, and facilitators of, implementing tumor screening programs for LS are presented. Specifically, respondents who indicated they had a tumor screening program at their center were asked about barriers to implementing the universal screening protocol, how barriers were overcome, and what advice they could offer for those centers currently planning a similar program. Respondents who specified at the start of the survey they did *not* have a universal tumor screening program at their center were directed to items exploring possible barriers to a screening program and what might be helpful in implementing universal tumor screening. Response options to close‐ended items were taken or adapted from Cohen.^21^

### 2.2 Survey administration

Surveys were hosted on SurveyMonkey between October 2016 and March 2017.^30^

### 2.3 Participant recruitment

All pathologists and genetic counselors in Canada were eligible to complete surveys, regardless of where they practiced or for how long. We were interested in the opinions of those with a tumor screening program for LS in their institution, but also those who did not have such a program. Thus, there were no exclusion criteria. The sampling frame consisted of genetic counselors and pathologists who were members of national professional organizations. A study invitation containing the survey link was advertised at least twice through professional disciplines’ regular communication channels (eg, newsletters, emails, social media posts). The Canadian Association of Pathology (CAP), the Canadian Association of Genetic Counselors (CAGC), and the Canadian Immunohistochemistry Quality Control all distributed study ads to their members. Research team members also sent personal study invitations containing the survey link through their networks. Four pathologists and three genetic counselors received personal study invites from team members and they were encouraged to share the link with colleagues.

### 2.4 Survey analysis

This was a descriptive study with no formal hypothesis. Rather, the goal was to describe barriers to implementing a tumor screening program and possible solutions from the perspective of those respondents who currently had a screening program at their centers. Those currently without a screening program were also asked to describe possible barriers and what might be helpful in establishing a program at their center. Descriptive statistics are used to describe responses to quantitative and qualitative items. Counts and percentages describe respondents’ endorsement of specific barriers and facilitators to implementing a tumor screening program for LS. All open‐ended questions were coded and analyzed according to the qualitative descriptive approach.^34^, ^35^ This is a naturalistic form of inquiry with no a priori theoretical assumptions about the data. Rather, data are presented in the language of participants and the end result is a comprehensive summary of responses. First, data were isolated according to question content (eg, barriers to a screening program, advice to others considering implementing a LS screening program). Two investigators (HE, GL) then independently read open‐ended responses and identified emerging themes across surveys. Following independent coding, these investigators met to discuss and finalize themes and representative quotes. Slight differences in codes and themes were resolved through discussion. This analysis was finally presented to the larger research team for discussion and approval.

## 3 RESULTS

One hundred and nineteen completed surveys were available for analysis (53 pathologists and 66 genetic counselors). Accurate response rates for the surveys could not be determined as there was no way of knowing exactly how many genetic counselors and pathologists saw the survey invite, opened the link, or started the survey. Survey invites were sent to 472 pathologists through the CAP, giving a crude response rate of approximately 11%. At the time of advertising, the CAGC confirmed there were 330 genetic counselors in their membership and on their mailing list, giving a crude response rate of 66/330 or 20%. Response to demographic items was poor across both groups. Just seven genetic counselors completed demographic information. All were female; four practiced in ON and three in Western Canada. Most were practicing for 11‐15 years; four specified “academic medical center” as their place of practice, three a community hospital, and one a diagnostic laboratory. Thirty‐two pathologists provided demographic data: two from Atlantic Canada, 14 from ON and QB, with 16 from Western Canada. There were roughly equal numbers of men and women (n = 16), in practice for more than 15 years (~29%). Almost half practiced in academic medical centers (41.5%), with approximately one quarter in community hospitals; the remainder identified private practice or diagnostic lab.

### 3.1 Perspectives of respondents with a tumor screening program at their centers

**Figure 1:**
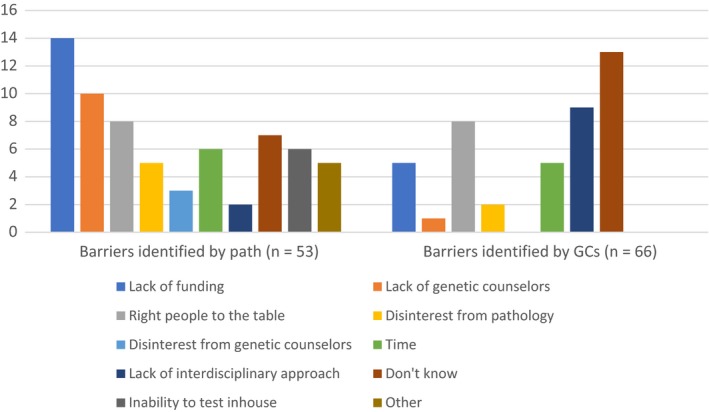
Responses (counts) of pathologists and genetics counselors to the item, “*What barriers did your centre face in implementing a screening protocol?*”

Respondents were asked to identify which barriers their center faced in implementing a tumor screening protocol (Figure 1). The most common response from pathologists was lack of funding, whereas genetic counselors most often indicated they did not know what the specific barriers were. Pathologists further endorsed a lack of genetic counselors as a significant barrier, while the genetic counselors identified the lack of an interdisciplinary approach as a common barrier. Both groups noted that not having the right people to the table was a barrier.

Respondents were asked to explain how they overcame some of the barriers in an open item. Table 1 summarizes key barriers and potential solutions that arose from the data. Similar barriers and solutions were endorsed by pathologists and genetic counselors and included having the right people to the table, funding issues, interdisciplinary teams, and awareness/interest mentioned most frequently. Access to funding for tumor screening programs was an oft‐cited barrier; respondents noted ongoing efforts to seek funding and the need for government support.

**Table 1:** Barriers to a tumor screening program for LS

Barrier to screening program	Possible solutions	Representative quotations Genetic counselors (n = 7); Pathologists (n = 11)
Ongoing access to adequate funding	Seek alternative sources of funding, including government	*The hospital has been willing to cover the IHC costs so far, but as costs continue to rise there are worries that there will be cut‐backs to our IHC budget*. –Pathologist *Found alternative ways to fund*. –Genetic counselor *I think much of the funding came through the cancer centre budget*. –Pathologist *A government commitment to fund downstream genetic testing*. –Pathologist *Overcome? Funding is an ongoing issue*. –Pathologist
Lack of interest/awareness	Raising awareness of tumor screening for LS to the right stakeholders	*Highlighting the importance of universal tumor testing and getting the right individuals on board. *–Genetic counselor
		*Continued discussion with pathologists on the need to test patients.* –Pathologist *Lack of interest in the program from the clinicians that directly interact with the patients (surgeons, oncologists).* –Pathologist
Lack of interdisciplinary approach	Building an interdisciplinary team	*When it came to our endometrial testing, we have a great group of people including pathology gyn/onc and genetics who routinely review these things. The right people are at the table. We need to establish the same multidisciplinary approach in colon.* –Genetic counselor *We are in the process of putting together a working group to look at this issue. We would like to see screening properly implemented…* –Pathologist
Identifying the right people to come to the table	Finding champions of tumor screening	*Getting informed and proactive individuals from health authority administrations to the table is always a challenge. Luckily, a champion of an epidemiologic approach to universal screening was hired as the new provincial Hereditary Cancer Programme and she was effective at implementing the program's follow up counselling and testing.* –Pathologist *We have good oncology groups*. –Pathologist *Initial meetings organized by genetic counselors. Original criteria [for screening] set by counselors*. –Pathologist

Respondents were provided with an opportunity to provide advice to centers that might be planning a LS screening program (Table 2). The most common themes that arose were related to how the program was developed and its ongoing structure and the different resources, training, and research that were available to support it.

**Table 2:** Advice for institutions planning a tumor screening program for Lynch Syndrome

Theme	Representative quotations Genetic counselors (n = 10); Pathologists (n = 7)
Program Development and Structure	*Definitely need buy‐in from pathology because that's where it all has to start. The gap in our process at this time is the referral getting to Genetics once the pathology report is available to the ordering MD I am quite sure that there are patients with IHC‐deficient tumours that are not referred to Genetics. If we had more staff/resources, I could propose that we receive the reports as well, however, this is not possible at this time. I attend Gyne‐Onc disease site team meetings every week, so I am able to remind and discuss cases with surgeons/oncologists regularly, so the referral process for gyne is good. I am unable to attend the GI disease site team meetings every week, mostly because they discuss at least 15 cases every week and do not have time to review genetic implications. As a result, I am sure there are patients that slip through the cracks.* –Genetic Counselor *Good quality control and participate in external IHC testing. *–Pathologist *Rather than cherry picking which cases to do, we found it easier to do all cases as a standard routine. It is much better and more cost effective to start with two antibody IHC screen (ie, PMS2 and MSH6 IHC) upfront and work from there.* –Pathologist *Be clear about the expectations of all parties involved and make sure you have a way to track results.* –Genetic Counselor *The only thing I could advise is that from start to finish, I think one person should be involved such as the genetic counselor – for the counseling consents, testing, results and any follow‐up testing.* –Genetic Counselor
Resources, research and training	*A screening program is very costly – need to have budget and hospital support. See if necessary lab tests are funded by the Provincial Ministry of Health. See if clinical genetics can accommodate the increased number of referrals*. –Pathologist *It's all in the data‐ many patients will be missed without proper implementation*. –Genetic Counselor *Compile epidemiological statistics on rates of MMR deletions and compare these to regional, national and international figures. *–Pathologist
	*Lots of resources from Heather Hampel and Lynch Syndrome Screening Network. *–Genetic Counselor *Education of pathologists. *–Pathologist

### 3.2 Perspectives of respondents without a tumor screening program at their centers

**Figure 2:**
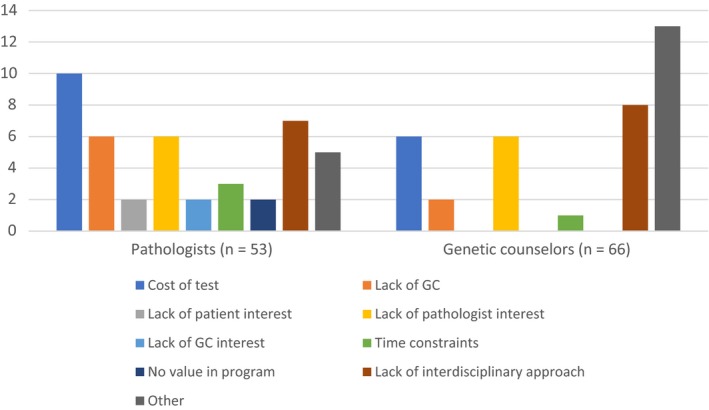
Responses (counts) of pathologists and genetics counselors to the question, “*Can you tell us why your centre does not do routine LS screening?*”

Respondents without a LS screening program at their center were asked to identify possible barriers. A list of nine different possibilities was presented, and respondents could choose as many they wished. The frequencies of each option selected are shown in Figure 2.

While counselors could not definitely say why screening protocols did not exist at their centers, they indicated a possible lack of knowledge about LS in general and noted there were no clear guidelines for tumor screening. One also noted the role of the host institution in implementing a screening program:

I think there are a number of interested parties (genetics, pathology, surgery, oncology) but it is the organization and decisions regarding initiating a routine screening program. There is also a need for education. –Genetic counselor

..this has been discussed, but I’m not sure what barrier(s) there have been to implementation. Testing is available, but to the best of my knowledge, is only performed when requested by a clinician. –Genetic counselor

Not clear on the criteria to use for this testing i.e., there is no provincial guideline dictating this testing be done if a patient is diagnosed under a specific age. –Genetic counselor

Lack of knowledge about hereditary genetic conditions beyond hereditary breast and ovarian cancer. –Genetic counselor

Pathologists most often identified the cost of the test as the reason for the lack of tumor screening at their center. One also noted the lack of provincial guidance: *Lack of endorsement and guidelines from [provincial cancer organization]*. Both groups also identified the lack of an interdisciplinary approach. Indeed, a lack of both resources and an interdisciplinary approach were identified by both genetic counselors and pathologists as the key barriers to implementing a tumor screening program for LS at their centers.

An interdisciplinary approach and increased funding are key. –Pathologist.

It is probably a combination of things, including lack of resources (money, trained persons and space). –Genetic counselor

Lack of communication between pathology, oncology and genetics. ‐Genetic counselor.

### 3.3 Potential facilitators for implementing a tumor screening program for LS

**Figure 3:**
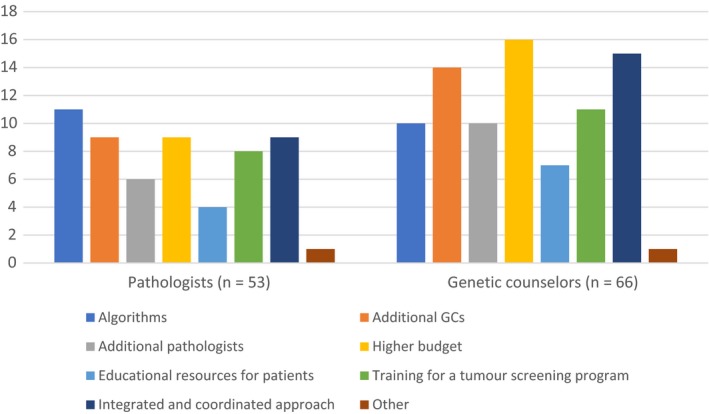
Responses (counts) by pathologists and genetics counselors to the question, “*What would be helpful to your centre if it attempted to implement routine screening for Lynch Syndrome?*”

Finally, respondents currently without a tumor screening program for LS were asked to choose what might facilitate their center's efforts to implement a routine screening program (Figure 3).

Despite repeated mentions from pathologists in open comments of the need for a higher budget, their most selected response was the need for Algorithms (Figure 3). A “higher budget” had as many responses as hiring “additional genetic counselors (GCs)” and using a “coordinated and integrated approach.” The most common responses from the genetic counselors were a “higher budget” followed by an “integrated and coordinated approach” and “additional GCs.”

## 4 DISCUSSION

Pathologists and genetic counselors identified numerous factors that were perceived to affect the implementation of a universal tumor screening program for LS.

A key barrier identified by all respondents was the paucity of resources available to design and maintain such a program, including a lack of dedicated funding and insufficient numbers of genetic counselors. Strong endorsement of the need for a coordinated, interdisciplinary approach to program planning with the right people at the table, including decision maker champions of tumor screening, was also observed.

Advice from respondents whose centers currently had a current screening program for LS revolved around careful design of program structure and ensuring adequate resources (funding, staff, training for providers) were available prior to program initiation. Indeed, adequate and ongoing funding, sufficient counseling staff, and having an integrated and coordinated interdisciplinary approach to screening were seen as key facilitators to implementing a screening program.

These barriers and facilitators are very consistent with the growing body of evidence on developing tumor screening programs for LS.^19^, ^20^, ^23^, ^32^ A coordinated and consistent approach to tumor testing is needed, with clear guidelines on the management of patients with LS and the roles and responsibilities of providers in the care pathway.^23^, ^26^, ^32^ Some respondents specifically noted the lack of provincial guidelines and this has been cited as a barrier in the literature.^32^

If a population benefit to tumor screening is to be realized, a broader coordinated approach to tumor testing is needed; lessons learned from provincial or state newborn screening programs could provide valuable information in this regard.^26^ Palter et al’s (2018) respondents noted that in order to ensure the consistency and quality of a provincial reflex tumor testing program, centralized oversight through a top‐down approach (ie, mandated) would be necessary.^23^ Given the heterogeneity of tumor testing programs in Canada, this approach may have merit.^22^, ^30^ However, it has been suggested that balancing a mandated program with bottom‐up input from all key stakeholders in the care pathway would likely help ensure program compliance and quality.^23^

A provincially or nationally mandated program might also help ensure ongoing funding, an oft‐cited concern by respondents and a common reported barrier.^21^, ^23^, ^32^ Bellcross et al (2012) suggested that a national screening program, rather than single institution protocols, could potentially reduce costs in the long term.^26^ By most measures, universal screening of newly diagnosed CRCs for LS is cost effective,^36^, ^37^ and is associated with improving cancer‐associated morbidity and mortality, as well as improving quality of life.^19^, ^38^ In Canada, the Canadian Agency for Drugs and Technology in Health released a health technology assessment about MMR deficiency testing for CRC patients and concluded that universal testing of all CRCs was one of the cost‐effective strategies for identifying high‐risk individuals and could also be more equitable.^39^ Practically, the upfront costs would likely be significant and those at the policy level would need to be convinced of the downstream cost savings.^23^, ^26^ However, evidence continues to emerge about the cost effectiveness of different screening approaches from real‐world clinical settings that should assist decision makers in implementing LS screening.^40^ In a comprehensive review of economic evaluations of LS screening, Di Marco and colleagues concluded^40^ that from a willingness to pay for health gains perspective, both universal and age‐targeted CRC‐based LS screening are cost effective; however, the choice of test for the initial screen (ie, IHC or MSI) and the choice of BRAF or MLH1 hypermethylation testing as follow‐up tests should depend on context‐specific factors such as local expertise and preference or available technologies.

Ultimately, the success of universal tumor screening programs hinges on the uptake of subsequent germline confirmatory testing by patients who screen positive, allowing their at‐risk family members to access appropriate screening and prevention options.^19^, ^41^ Some respondents observed that regular tracking of data and education for all clinical stakeholders, as well as patients and families, would be essential if a universal tumor screening program was to be successful. These results and others^16^, ^23^, ^26^ highlight the need to build awareness of LS and tumor screening with ongoing continued medical education for all stakeholders in the care pathway. Onsite champions could assist in this regard, and in this study, respondents without such a champion highlighted the difficulty of convincing medical staff of the necessity of such a program.

Genetic counseling resources in particular are critical to ensure patient and family education^23^ and many of the current study's respondents cited the lack of genetic counselors as a barrier to program implementation. Indeed, programs with high levels of genetic counseling involvement reported better patient adherence to follow‐up protocols and better notification of positive tumor screens for LS.^27^ Thus, sufficient resources will be required to implement the necessary education programs for all stakeholders, including patients and families.

There are several limitations to the study. First, it included only two stakeholder provider groups. While they are key stakeholders in a tumor screening program, the views and experiences of surgeons and oncologists who care for cancer patients and would therefore be involved in a tumor screening process are missing. Thus, this sample is not representative of all providers working within tumor screening programs in Canada. Further, the sampling strategy limited the participation in the study to those genetic counselors and pathologists who were members of national professional organizations. It is unknown how many of these providers are not members of such groups, nor it is known how their opinions and experiences might differ from members. While there is no reason to believe their perceptions of tumor screening for LS would be significantly different than current respondents, this cannot be known with certainty. Thus, the study's sample is not representative of the Canadian population of genetic counselors and pathologists. Survey response rates were low, and the online survey platform did not allow an accurate calculation of response rate. We could not adequately describe our sample as there was high non‐response to demographic items. In fact, responses to demographic items revealed limited geographic scope of participation of providers; all provinces were not equally represented. This could suggest that study respondents were from areas more aware of tumor testing for LS or more interested in screening programs compared to the larger population of providers in Canada, thus limiting the generalizability of results. The research team made many efforts to advertise the survey through national organizations and professional networks. Online survey methods may have limited acceptability or the topic was perceived as not relevant to these professional groups. Different recruitment methods will be needed in future research if study findings are to be reflective of national practice.

Despite these limitations, results add to the literature on barriers and facilitators of implementing a tumor screening program for LS. Findings are consistent with the growing body of lessons learned and should inform the planning of tumor screening programs. Identifying the right stakeholders, finding champions of tumor screening, building an interdisciplinary and coordinated approach to screening, ensuring adequate education of all stakeholders, and finding initial and sustained funding were all identified as important factors to consider. Variability in the existence of tumor screening programs across Canada also suggests the need for national standards and guidance on tumor screening for LS.^22^, ^30^

## CONFLICT OF INTEREST

The authors declare no conflict of interest.

## AUTHOR CONTRIBUTIONS

Dicks and Etchegary conceived of the study, with specific input from all authors on the final protocol. Kao, Pullman, MacMillan, and Simmonds helped with recruitment. Dicks, Etchegary, and Logan analyzed the data and wrote the article. All authors reviewed the article and approved the final submission.

## DATA AVAILABILITY STATEMENT

The data that support the findings of this study are available from the corresponding author upon reasonable request.
